# Kidney function and multiple hemostatic markers: cross sectional associations in the multi-ethnic study of atherosclerosis

**DOI:** 10.1186/1471-2369-12-3

**Published:** 2011-01-26

**Authors:** Ruth Dubin, Mary Cushman, Aaron R Folsom, Linda F Fried, Walter Palmas, Carmen A Peralta, Christina Wassel, Michael G Shlipak

**Affiliations:** 1 Dept. of Medicine, Division Nephrology, University of California San Francisco, 521 Parnassus Avenue, Room C443, San Francisco, CA 94143-0532, USA; 2 Fletcher Allen Health Care, Thrombosis and Hemostasis Program, Hematology/Oncology Clinic, 111 Colchester Avenue, Burlington, VT 05401, USA; 3 Division of Epidemiology & Community Health, 1300 South Second Street, Suite 300 Minneapolis, MN 55454, USA; 4 Division of Nephrology, 7E121 VA Pittsburgh Healthcare System, University Drive Center, Pittsburgh, PA, 15240, USA; 5 Dept. of Internal Medicine, Columbia University, Presbyterian Hospital, Room 9E-107, 622 West 168th St., New York, NY 10032, USA; 6 Dept. of Family and Preventive Medicine, UCSD School of Medicine, 9500 Gilman Drive #0965, La Jolla CA 92093, USA; 7 Division of General Internal Medicine, San Francisco VA Medical Center, 4150 Clement St. Rm. 111A1, San Francisco, CA 94143, USA

## Abstract

**Background:**

Defined as estimated glomerular filtration rate (eGFR) < 60 ml/min/1.73 m2, chronic kidney disease (CKD) is strongly and independently associated with cardiovascular and overall mortality. We hypothesized that reduced kidney function would be characterized by abnormalities of hemostasis.

**Methods:**

We tested cross-sectional associations between (eGFR) and multiple hemostatic markers among 6751 participants representing a broad spectrum of kidney function in the Multi-Ethnic Study of Atherosclerosis (MESA). Kidney function was measured using cystatin C (eGFRcys) or creatinine, using CKD Epidemiology Collaboration (eGFRcr). Hemostatic markers included soluble thrombomodulin (sTM), soluble tissue factor (sTF), D-Dimer, von Willebrand factor (vWF), factor VIII, plasmin-antiplasmin complex (PAP), tissue factor pathway inhibitor (TFPI), plasminogen activator inhibitor-1 (PAI-1), and fibrinogen. Associations were tested using multivariable linear regression with adjustment for demographics and comorbidities.

**Results:**

In comparison to persons with eGFRcys >90 ml/min/1.73 m^2^, subjects with eGFRcys < 60 ml/min/1.73 m^2 ^had adjusted levels of sTM, sTF, D-Dimer, PAP, Factor VIII, TFPI, vWF and fibrinogen that were respectively 86%, 68%, 44%, 22%, 17%, 15%, 12% and 6% higher. Subjects with eGFRcys 60-90 ml/min/1.73 m^2 ^had adjusted levels that were respectively 16%, 14%, 12%, 6%, 6%, 6%, 11% and 4% higher (p < 0.05 for all). Percent differences were not significantly different when groups were categorized by eGFRcr.

**Conclusions:**

Throughout a broad spectrum of kidney function, lower eGFR was associated with higher levels of hemostatic markers. Dysregulation of hemostasis may be a mechanism by which reduced kidney function promotes higher cardiovascular risk.

## Background

Defined as estimated glomerular filtration rate (eGFR) <60 ml/min/1.73 m^2^, chronic kidney disease (CKD) stage 3 to 5 [1] is an established risk factor for cardiovascular morbidity and mortality [2,3]. This association begins in the pre-clinical stages of kidney disease, when kidney function may be better estimated by cystatin C (eGFRcys) [4-6]. The mechanisms underlying the association of CKD with cardiovascular morbidity and mortality are inadequately understood. In multiple cohorts with and without CKD, the associations of lower eGFR with higher cardiovascular risk persist despite adjustment for traditional risk factors and inflammation [7-10], prompting investigation into novel pathways between renal and cardiovascular disease (CVD).

Atherosclerosis represents a pernicious interplay between systems of inflammation and coagulation [11]. Multiple investigators have reported an association between creatinine-based CKD and elevated levels of inflammatory and thrombotic markers [12,13]. In studies from the Health, Aging and Body Composition Study and the Multi-Ethnic Study of Atherosclerosis (MESA), subjects with mildly reduced kidney function measured with cystatin C had only modest elevations in inflammatory markers [13-16]. Furthermore, the positive association of cystatin C with cardiovascular and overall mortality is only partially attenuated by adjustment for C-reactive protein (CRP), interleukin-6 (IL-6) or TNF-α [5,6]. However, whether or not dysregulation of hemostasis contributes to the association of CKD and CVD has not been well evaluated.

We designed these analyses to explore the associations of reduced kidney function, as measured by eGFRcys and CKD Epidemiology Collaboration (eGFRcr), with hemostatic factors in an ethnically diverse cohort free of clinical cardiovascular disease.

## Methods

### Subjects

We conducted a cross-sectional analysis of 6751 participants in the Multi Ethnic Study of Atherosclerosis (MESA) at the baseline visit (July 2000 to July 2002). Soluble thrombomodulin (sTM), soluble tissue factor (sTF) and tissue factor pathway inhibitor (TFPI) were measured in a random sample of 1000 MESA participants; the other markers were measured in nearly the whole cohort. MESA is a cohort study sponsored by the National Heart, Lung, and Blood Institute aimed to improve understanding of subclinical cardiovascular disease and its progression in a multiethnic cohort. Details on recruitment and design have been published [17]. Briefly, MESA recruited 6,814 men and women between the ages of 45 and 84 years, who were free of cardiovascular disease and self-identified as white, African American, Hispanic, or Chinese-American. Subjects were recruited from Baltimore City and Baltimore County, Maryland; Chicago, Illinois; Forsyth County, North Carolina; Los Angeles County, California; northern Manhattan and the Bronx, New York; and St. Paul, Minnesota, between July 2000 and August 2002. Individuals were excluded if they had physician-diagnosed heart attack, angina, heart failure, stroke or transient ischemic attack, or atrial fibrillation; if they had undergone coronary artery bypass grafting, angioplasty, valve replacement, or pacemaker insertion; or if they weighed > 300 pounds (>136 kg). The institutional review boards at all participating centers approved the study, and all participants gave informed consent.

### Primary predictors

Cystatin C was measured in plasma using a BNII nephelometer on plasma specimens (Siemens Latex N-Latex cystatin C assay). Estimated glomerular filtration rate by cystatin C (eGFRcys) was calculated using the formula: 76.7 × cys C ^-1.19^. This formula was developed from pooled data from several cohorts with eGFR measured by iothalamate [18]. Serum creatinine was measured using colorimetry with a Johnson & Johnson Vitros 950 analyzer (Johnson & Johnson Clinical Diagnostics Inc., Rochester, NY). Creatinine levels were calibrated to the Cleveland Clinic standard (0.9954*Cr + 0.0208) [19]. The eGFRcr equation was used to calculate creatinine - based eGFR (eGFRcr) [20].

### Primary outcomes

Soluble thrombomodulin (sTM) was measured by enzyme immunoassay using a monoclonal antibody to TM as the capture antibody (Asserachrom Thrombomodulin, Diagnostica Stago; Asnières-sur-Seine, France, CV 12%). Soluble tissue factor (sTF) was measured by an enzyme-linked immunoassay that employs an anti-TF monoclonal capture antibody (Imubind Tissue Factor ELISA Kit, American Diagnostica, Inc.; Stamford, CT, CV 14.6%). Total tissue factor pathway inhibitor (TFPI) was measured by enzyme-linked sandwich ELISA using a polyclonal anti-TFPI antibody as the capture antibody (Imubind Total TFPI ELISA Kit, American Diagnostica, Inc.; Stamford, CT). Intra-assay and inter-assay analytical CVs range from 6.2-7.1% and 5.5-7.3%, respectively. Fibrin fragment D-dimer was measured using an immuno-turbidimetric assay (Liatest D-DI; Diagnostica Stago, Parsippany, NJ) on the Sta-R analyzer (Diagnostica Stago, Parsippany, NJ, CV 8%). Plasmin-antiplasmin complex (PAP) was measured in an assay that detects only plasmin in complex with α_2_-antiplasmin, and not free plasmin or α_2_-antiplasmin, CV 1.7%. von Willebrand factor was measured by an immunoturbidometric assay on the Sta-R analyzer (Diagnostica Stago, Parsippany, NJ). The intra-assay and inter-assay analytical CV's are 3.7% and 4.5%, respectively. Factor VIII coagulant activity was determined by measuring the clotting time of a sample in factor VIII deficient plasma in the presence of activator (STA-Deficient VIII; Diagnostica Stago, Parsippany, NJ). Fibrinogen was measured using a BNII nephelometer (N Antiserum to Human Fibrinogen; Siemens) with intra-assay and inter-assay analytical CV's of 2.7% and 2.6%, respectively. Plasminogen activator inhibitor-1 (PAI-1) was measured by a two-site ELISA [21].

### Variables of interest

Blood pressure measurements were obtained by using an automated blood pressure device (DINAMAP PRO 100 monitor; General Electric Healthcare) following JNC guidelines [22]. Hypertension was defined as self-report of physician diagnosis, use of anti-hypertensive agents, or SBP > = 140, or DBP > = 90. Diabetes was defined by fasting glucose > = 126 or the use of hypoglycemic medication or insulin. Tobacco use was defined by current smoking. Height and weight were measured with participants wearing light clothing and no shoes. Body mass index was calculated as weight (kg)/height (m)^2^. Fasting blood was collected and stored at -70 F (21.11 C) until analyzed for total and high density lipoprotein cholesterol, triglycerides and glucose. Low density lipoprotein cholesterol was calculated by using the Friedewald equation.

### Statistical analysis

We compared sociodemographic and anthropometric factors as well as comorbidities among subjects with eGFRcys > 90, 60-90 and < 60 ml/min/1.73 m^2 ^using ANOVA, Kruskal-Wallis test, or chi-square tests as appropriate. Using multivariable linear regression, we studied the associations between continuous eGFR and each of the seven biomarkers, using both eGFRcys and eGFRcr. Each biomarker was natural log transformed, and beta coefficients were transformed into percent differences using (e^β-1)*100 to allow comparison across outcomes. In additional analyses we defined eGFR groups as >90, 60-90 and <60 ml/min/1.73 m^2^, and we compared the percent difference in each biomarker with eGFR > 90 as the reference. The fully adjusted models included age, race/ethnicity (white, black, Hispanic, Chinese), gender, annual income, study site, current smoking, current alcohol use, BMI, DM, HTN, statin use, ACE-I, LDL cholesterol, HDL cholesterol, triglycerides, fasting glucose, and ln(albumin/creatinine). All models assumptions for linear regression, including linearity, homoscedasticity and normality, were checked. Analyses were preformed in SAS V 9.1.3 (SAS Institute, Cary, NC), and p < 0.05 was considered as the threshold for statistical significance.

## Results

### Participants' characteristics

Among the 6751 participants, the mean age was 62 years; 53% were women; 38% were white, 27% black, 22% Hispanic, and 12% Chinese-American. The prevalences of diabetes and hypertension were 14% and 45%, respectively. Average eGFRcys was 92 ml/min/1.73 m^2 ^and average eGFR by eGFRcr was 79 ml/min/1.73 m^2 ^. Compared to those without CKD, subjects with CKD with eGFRcys <60 ml/min/1.73 m^2 ^were older, had a higher prevalence of diabetes and hypertension, higher rate of ACE-I and statin use, higher fasting glucose, triglycerides and urinary albumin to creatinine ratio, lower HDL and LDL, and lower rate of alcohol use. (Table 1)

**Table 1:** Demographic and clinical characteristics of MESA participants by groups of eGFRcys, 2000-2002

	eGFRcys <60 (n = 376)	eGFRcys 60-90 (n = 2746)	eGFRcys >90 (n = 3629)	P Value*
**Age**, years†	71 ± 9	66 ± 10	59 ± 9	<0.001
**Female**, n(%)	185 (49)	1131 (48)	2066 (57)	<0.001
**Race/Ethnicity**, n(%)				<0.001
Caucasian	165 (44)	1151 (42)	1286 (35)	
African-American	103 (27)	715 (26)	1048 (29)	
Hispanic	72 (19)	617 (23)	795 (22)	
Chinese-American	36 (10)	263 (10)	500 (14)	
**Current Smoker**, n(%)	50 (13)	386 (14)	440 (12)	0.08
**Current Alcohol Use**, n(%)	169 (45)	1457 (53)	2087 (58)	<0.001
**BMI**, kg/m^2^	30 ± 6	29 ± 6	28 ± 5	
**Prevalent Diabetes**, n(%)	94 (25)	401 (15)	464 (13)	<0.001
**Prevalent Hypertension**, n(%)	272 (72)	1451 (53)	1310 (36)	<0.001
**ACE Inhibitor Use**, n(%)	113 (30)	501 (18)	410 (11)	<0.001
**Statin Use**, n(%)	77 (21)	465 (17)	459 (13)	<0.001
**Fasting Plasma Glucose**, mg/dl	111 ± 34	105 ± 29	104 ± 32	<0.001
**Triglycerides**, mg/dl§	131 (93, 180)	126 (88, 179)	107 (77, 157)	<0.001
**HDL Cholesterol**, mg/dl	47 ±14	49 ± 14	53 ±15	<0.001
**LDL Cholesterol**, mg/dl	114 ± 35	116 ± 6	118 ± 31	0.01
**Urinary Creatinine**, mg/dl‡	96 (65, 142)	111 (66, 163)	106 (55, 162)	0.001
**Urine Albumin/Creatinine**, mg/g	10 (5, 47)	6 (3, 12)	5 (3, 10)	<0.001
**Serum Creatinine**, mg/dl	1.4 ± 0.8	1.0 ± 0.2	0.9 ± 0.2	<0.001
**eGFR Creatinine**, ml/min/1.73 m^2^	52 ± 17	73 ± 14	87 ± 14	<0.001
**eGFR Cystatin**, ml/min/1.73 m^2^	50 ± 10	78 ± 8	108 ± 15	<0.001

*P-values obtained using ANOVA

†Unless otherwise noted, Mean (SD)

‡Median (Quartile 1, Quartile 3)

### Variation by race/ethnicity and correlations among biomarkers

We analyzed levels of hemostatic markers in different groups of race/ethnicity. sTM showed significant heterogeneity by race/ethnicity: Caucasians and Hispanics had significantly higher sTM than African-Americans and Chinese. Levels of vWF, and fVIII were significantly higher in African-Americans, and fibrinogen was higher in African Americans and Hispanics. (Table 2) As expected, many of the hemostatic biomarkers were highly correlated. vWF and factor VIII were positively correlated (r = 0.66); PAP and PAI-1 were inversely correlated, ( r = -0.48). D-Dimer had moderate correlations (r = 0.23-0.34) with VWF, factor VIII, PAP and fibrinogen. Fibrinogen had moderate correlations (r = 0.23-0.38) with vWF, factor VIII, PAP, and TFPI (p < 0.05 for all). No other strong correlations were observed. (Table 3)

**Table 2:** Levels of hemostatic markers by racial/ethnic group*

	All	Caucasian	African-American	Chinese	Hispanic	P Value**
**Soluble Thrombomodulin**, ng/ml	35 (16, 45)	36 (27, 47)	32 (23, 42)	31 (23, 40)	37 (27, 46)	<0.001
**Soluble Tissue Factor**, pg/ml	106 (68, 156)	107 (66, 149)	106 (72, 175)	97 (65, 169)	108 (68, 155)	0.53
**D-dimer**, ug/ml	0.20 (0.13, 0.37)	0.20 (0.13, 0.35)	0.25 (0.15, 0.44)	0.18 (0.10, 0.30)	0.23 (0.13, 0.41)	<0.001
**von Willebrand Factor**, %	130 (99, 169)	124 (95, 166)	144 (113, 199)	130 (96, 168)	127 (96, 166)	<0.001
**Factor VIII**, %	93 (73, 120)	90 (70, 115)	100 (77, 131)	93 (73, 115)	91 (74, 117)	<0.001
**Plasmin Antiplasmin**, nM	4.4 (3.4, 5.6)	4.4 (3.4, 5.6)	4.8 (3.8, 6.2)	3.9 (3.1, 4.9)	4.3 (3.4, 5.4)	<0.001
**Tissue Factor Pathway Inhibitor**, ng/ml	48 (38, 57)	49 (38, 57)	48 (40, 58)	40 (33, 50)	49 (40, 58)	<0.001
**Plasminogen Activator Inhibitor-1**, ng/ml	19 (10, 36)	19 (9, 34)	17 (8, 33)	24 (12, 39)	21 (11, 40)	0.02
**Fibrinogen**, mg/dl	338 (295, 389)	328 (286, 373)	353 (304, 406)	323 (287, 366)	352 (308, 401)	<0.001

*Levels displayed as median (Quartile 1, Quartile 3).

**By Kruskal-Wallis test for difference among racial/ethnic groups.

**Table 3:** Spearman correlations among hemostatic markers

	sTM	STF	D-dimer	vWF	Factor VIII	PAP	TFPI	PAI-1	Fib
**sTM**	--								
**STF**	0.15†	--							
**D-dimer**	0.05	0.04	--						
**vWF**	0.15†	0.09**	0.25†	--					
**Factor VIII**	0.10**	0.02	0.23†	0.66†	--				
**PAP**	-0.03	0.15†	0.34†	0.25†	0.21†	--			
**TFPI**	0.21†	0.08*	0.14†	0.16†	0.16†	0.10**	--		
**PAI-1**	0.08*	-0.08*	0.02	0.05	0.07*	-0.48†	0.13†	--	
**Fib**	0.03	0.07*	0.29†	0.26†	0.24†	0.38†	0.23†	0.08*	--

*0.01-<0.05

**0.001-<0.01

†<0.001

### Associations between eGFR and biomarkers

We analyzed the associations between continuous measures of eGFR measured by both eGFRcys and eGFRcr equations and the seven biomarkers. In models adjusted for sociodemographics and comorbidities, sTM and sTF had strong correlations with eGFR by either method. For every 10 ml/min/1.73 m^2 ^lower eGFRcys, sTM was 7% higher (p < 0.001) and sTF was 6% higher (p < 0.001). D-Dimer, vWF, Factor VIII, PAP and TFPI had moderate associations with eGFR using either method. D-Dimer was 5% higher, vWF was 3% higher, and the other three were 2% higher for every 10 ml/min/1.73 m^2 ^lower eGFRcys (p < 0.001 for all). PAI-1 was associated with eGFRcys but not eGFRcr; fibrinogen had a weak but statistically significant association with both GFR estimates. (Table 4)

**Table 4:** Cross-sectional associations of eGFR measures with hemostatic markers in MESA, 2000-2002: percent difference in hemostatic marker per 10 ml/min/1.73 m^2 ^decrement in eGFR*

	eGFRcys, % (95% CI)	eGFRcr, % (95% CI)
**Soluble Thrombomodulin**	7.4 (5.9, 8.8)	8.2 (6.3, 10.1)
**Soluble Tissue Factor**	6.1 (3.6, 8.5)	7.6 (4.4, 10.6)
**D-dimer**	4.6 (3.5, 5.7)	4.7 (3.3, 6.1)
**Von Willebrand Factor**	2.8 (1.4, 4.2)	2.8 (0.93, 4.6)
**Factor VIII**	2.2 (2.7, 1.8)	1.8 (2.5, 1.2)
**Plasmin-Antiplasmin Complex**	2.2 (1.7, 2.7)	3.2 (2.6, 3.8)
**Tissue Factor Pathway Inhibitor**	2.1 (1.2, 3.1)	1.4 (0.2, 2.6)‡
**Plasminogen Activator Inhibitor-1**	4.7 (2.0, 7.4)	1.3 (-2.3, 5.1)§
**Fibrinogen**	1.0 (0.74, 1.2)	1.0 (0.64,1.3)

*Note: Models adjusted for age, race, gender, SES (income), site, current smoking, current alcohol, BMI, prevalent DM, prevalent HTN, statin use, ACE-I, LDL, HDL, TG, fasting glucose, and ln(albumin/creatinine). All p values are <0.001 unless otherwise noted.

‡p < 0.05

§Non-significant

**Figure 1:**
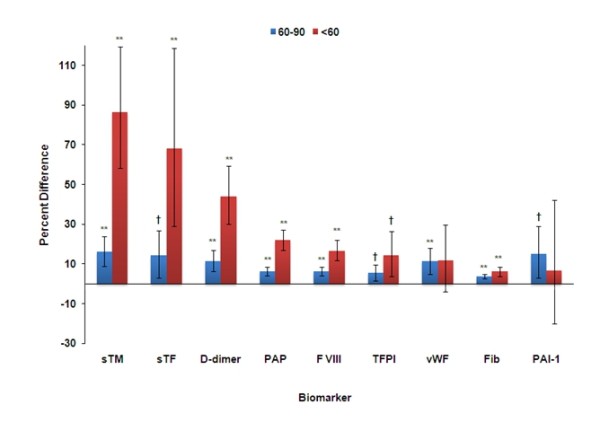
Percent Difference in Biomarker in Groups of eGFR cystatin in MESA*. * Models adjusted for age, race, gender, SES (income), site, current smoking, current alcohol, BMI, prevalent DM, prevalent HTN, statin use, ACE-I, LDL, HDL, TG, fasting glucose, and ln(albumin/creatinine); eGFR cystatin > 90 is the reference group ** p≤0.001 † p≤0.05

In addition, we calculated percent differences for each marker between subjects grouped by eGFR, using eGFR > 90 ml/min/1.73 m^2 ^as the reference. The group with eGFRcys <60 ml/min/1.73 m^2 ^had an 86% higher sTM level, 68% higher sTF, 44% higher D-Dimer, 12% higher vWF, 17% higher Factor VIII, 22% higher PAP, 15% higher TFPI, 6.5% higher PAI-1, and 6.3% higher fibrinogen. Participants with eGFR 60-90 ml/min/1.73 m^2 ^had significantly higher levels of all biomarkers ranging from 16% for sTM to 3.8% for fibrinogen. (Figure 1)

## Discussion

In a large, multiethnic cohort free of cardiovascular disease and with a spectrum of kidney function ranging from normal to moderate CKD, even small decrements in eGFR were associated with significantly higher levels of sTM, sTF, D-Dimer, vWF, factor VIII, PAP and TFPI. Our results suggest that dysregulation of hemostasis could play an important pathologic role in CKD. In particular, these associations may help to explain the consistently strong and independent associations of cystatin C with cardiovascular and overall mortality, despite adjustment for known risk factors including CRP [5], IL-6 and TNF-α [6].

Soluble thrombomodulin and soluble tissue factor had the largest associations with eGFRcys. sTM is a receptor for thrombin found on vascular and lymphatic endothelial cells. Injured endothelial cells release sTM into the circulation, where it binds thrombin, inhibiting it from forming fibrin and activating platelets. sTM has additional anti-thrombotic activity as a cofactor for the thrombin-catalyzed activation of protein C [23]. Elevated sTM is associated with lower eGFR in CKD and end stage renal disease ESRD [24,25], and our results extend these findings to a broader spectrum of eGFR. The association of sTM and cardiovascular disease varies by patient population: in healthy individuals, lower sTM predicts coronary heart disease (CHD), but in patients with established CHD, higher sTM levels are associated with recurrence of CHD and with higher mortality [26]. Whether higher levels of sTM are associated with CVD or mortality in our cohort would require further investigation.

Tissue factor (TF) initiates the extrinsic coagulation cascade. Smooth muscle cells, adventitial fibroblasts, and pericytes constitutively express TF, but endothelial cells express TF only after injury [27]. TF has a prominent role in atherosclerotic plaque rupture and acute coronary syndrome (ACS). TF is concentrated in the core of the atherosclerotic plaque; when the plaque ruptures, TF comes into contact with blood, binds factor VII and initiates the extrinsic coagulation cascade [28]. Higher levels of sTF were reported previously in CKD as compared to healthy subjects [29]. Epidemiological studies of sTF and CVD occurrence in healthy individuals are lacking, but in patients with ACS, high levels of sTF on admission are associated with poor outcome [30]. To our knowledge this paper is the first to report an association between higher sTF and lower eGFR in patients without CKD. Whether high levels of sTF contribute to the high cardiovascular mortality of persons with kidney disease is unknown.

D-Dimer, vWF, Factor VIII, and PAP showed moderate associations with lower eGFRcys. Elevated levels of D-Dimer are associated with lower eGFR [12] and CVD [31] across the spectrum of eGFR. vWF is increased in subjects with CKD [32], and it is considered to be a marker for endothelial dysfunction [33] and CVD risk [34,35]. Factor VIII is increased in subjects with CKD [12,32]. In the Atherosclerosis Risk in Communities (ARIC) Study, factor VIII was associated with progression of CKD and with fatal MI [36]. In MESA, Factor VIII was associated with overall but not cardiovascular mortality [37]. Plasmin, an enzyme that increases fibrinolysis, is inactivated when complexed with a2-antiplasmin to form PAP [11]. Subjects with CKD have higher levels of PAP than subjects without CKD [12]. In a cohort with CAD, PAP was associated with higher CVD risk [38]; in the Cardiovascular Health Study (CHS), PAP was associated with incident myocardial infarction [39].

Several of the hemostatic markers evaluated in this study are strong risk factors for venous thomboembolic disease, including D-dimer, factor VIII, and vWF [40]. Recent epidemiological studies have linked CKD with risk of venous thrombosis [41,42]. Thus, our findings may be applicable to both arterial and venous thrombosis, and these associations may extend into higher levels of kidney function than previously appreciated.

There are several possible mechanisms to explain the association of lower eGFR and higher levels of hemostatic factors. A direct effect of decreased renal clearance may explain an increase in levels of smaller molecular weight hemostatic markers such as sTF and sTM, as these may be filtered at the glomerulus. Fragments of sTM ranging from 28 to 108 kDa can be detected in blood and urine [43]. Soluble tissue factor has a MW of 29 kDa. Urinary tissue factor, with MW of 34 kDa, is elevated in glomerulonephritis and various malignancies, but it is thought to originate from the kidneys, rather than being blood borne and filtered at the glomerulus [44]. Whether sTF and sTM are filtered at the glomerulus would have to be tested in animal studies. Given the higher molecular weight of D-Dimer, vWF, factor VIII and PAP, it seems unlikely that these markers are elevated as a direct result of decreased renal clearance. It is possible that elevations in these markers relate to processes initiated by smaller molecules. Conversely, kidney dysfunction may generate a thrombotic milieu indirectly through electrolyte or acid-base abnormalities, which may alter activities of enzymes involved in coagulation. Inflammation associated with lower eGFR may cause elevations in markers that are acute phase reactants. Alternatively, microvascular disease may promote the development of CKD, and in parallel, may cause elevations in these hemostatic biomarkers; in this scenario, the markers would be neither cause nor effect of lower eGFR.

A strength of this study is the unique setting, as MESA is a large, well-characterized cohort including four major race/ethnic groups. The use of cystatin C to measure eGFR increases our ability to detect associations of biomarkers across a broader spectrum of kidney function. However, there are important limitations to our study. As is typical for most large epidemiological studies, we did not use a gold standard measure of eGFR, such as iothalamate or iohexol clearance. Due to the cross-sectional design, we cannot ascertain the direction of association; it is equally possible that increased levels of thrombotic markers causes decreased eGFR, or that decreased eGFR causes the elevated markers. Finally, residual confounding may exist in our analysis, which may account for some of our findings, but would be less likely to account for the strong associations of eGFRcys with sTM and sTF.

## Conclusions

In summary, lower eGFR was significantly associated with higher levels of sTM, sTF, D-Dimer, PAP, Factor VIII, TFPI, vWF and fibrinogen in subjects with and without CKD. Increased levels of smaller molecular weight molecules such as sTM and sTF may be a direct result of decreased renal clearance. Elevations of larger molecules may represent increased fibrinolytic activity and higher clot burden of a thrombotic milieu induced by declining kidney function. Alternatively, the atherosclerotic process may differ fundamentally in persons with lower eGFR. Further investigations may lead to a better understanding of the mechanisms underlying these associations, as well as the role of these associations in predicting cardiovascular disease in subjects with elevated cystatin C.

## Competing interests

The authors declare that they have no competing interests.

## Authors' contributions

RD drafted and revised the paper, and gave input into statistical analysis. MC helped to draft the manuscript and provided advice on hemostatic factors. AF helped draft the manuscript and provided advice on interpreting the data. LF helped draft the manuscript and provided advice on interpreting the data. WP helped draft the manuscript and provided advice on interpreting the data. CP helped draft the manuscript and provided advice on interpreting the data. CW performed statistical analysis. MS sponsored and conceived of the project, helped revise the paper, and gave input into statistical analysis. All authors read and approved the final manuscript.

## Pre-publication history

The pre-publication history for this paper can be accessed here:

http://www.biomedcentral.com/1471-2369/12/3/prepub

## References

[B1] KDOQI Definition and Classification of Stages of Chronic Kidney Disease 2002 http://www.kidney.org/professionals/kdoqi/guidelines_ckd/p4_class_g2.htm

[B2] Coresh J Astor B Sarnak MJ Evidence for increased cardiovascular disease risk in patients with chronic kidney disease Curr Opin Nephrol Hypertens 2004 13 1 73 81 10.1097/00041552-200401000-00011 15090863

[B3] Schiffrin EL Lipman ML Mann JF Chronic kidney disease: effects on the cardiovascular system Circulation 2007 116 1 85 97 10.1161/CIRCULATIONAHA.106.678342 17606856

[B4] Shlipak MG Katz R Sarnak MJ Fried LF Newman AB Stehman-Breen C Cystatin C and prognosis for cardiovascular and kidney outcomes in elderly persons without chronic kidney disease Ann Intern Med 2006 145 4 237 46 10.7326/0003-4819-145-4-200608150-00003 16908914

[B5] Shlipak MG Sarnak MJ Katz R Fried LF Seliger SL Newman AB Cystatin C and the risk of death and cardiovascular events among elderly persons N Engl J Med 2005 352 20 2049 60 10.1056/NEJMoa043161 15901858

[B6] Shlipak MG Wassel Fyr CL Chertow GM Harris TB Kritchevsky SB Tylavsky FA Cystatin C and mortality risk in the elderly: the health, aging, and body composition study J Am Soc Nephrol 2006 17 1 254 61 10.1681/ASN.2005050545 16267155

[B7] Stenvinkel P Carrero JJ Axelsson J Lindholm B Heimburger O Massy Z Emerging biomarkers for evaluating cardiovascular risk in the chronic kidney disease patient: how do new pieces fit into the uremic puzzle? Clin J Am Soc Nephrol 2008 3 2 505 21 10.2215/CJN.03670807 PMC6631093 18184879

[B8] Cheung AK Sarnak MJ Yan G Dwyer JT Heyka RJ Rocco MV Atherosclerotic cardiovascular disease risks in chronic hemodialysis patients Kidney Int 2000 58 1 353 62 10.1046/j.1523-1755.2000.00173.x 10886582

[B9] Massy ZA Taupin P Jungers P Landais P Prediction model of coronary heart disease in patients with chronic kidney disease: role of plasma fibrinogen as a new prognostic variable Prilozi 2005 26 2 63 77 16400230

[B10] Spiegel DM Raggi P Smits G Block GA Factors associated with mortality in patients new to haemodialysis Nephrol Dial Transplant 2007 22 12 3568 72 10.1093/ndt/gfm424 17617651

[B11] Hellenthal FA Buurman WA Wodzig WK Schurink GW Biomarkers of AAA progression. Part 1: extracellular matrix degeneration Nat Rev Cardiol 2009 6 7 464 74 10.1038/nrcardio.2009.80 19468292

[B12] Shlipak MG Fried LF Stehman-Breen C Siscovick D Newman AB Chronic renal insufficiency and cardiovascular events in the elderly: findings from the Cardiovascular Health Study Am J Geriatr Cardiol 2004 13 2 81 90 10.1111/j.1076-7460.2004.02125.x 15010654

[B13] Shlipak MG Katz R Cushman M Sarnak MJ Stehman-Breen C Psaty BM Cystatin-C and inflammatory markers in the ambulatory elderly Am J Med 2005 118 12 1416 10.1016/j.amjmed.2005.07.060 16378798

[B14] Keller C Katz R Sarnak MJ Fried LF Kestenbaum B Cushman M Inflammatory biomarkers and decline in kidney function in the elderly: the Cardiovascular Health Study Nephrol Dial Transplant 2010 25 1 119 24 10.1093/ndt/gfp429 PMC2910326 19734138

[B15] Keller CR Odden MC Fried LF Newman AB Angleman S Green CA Kidney function and markers of inflammation in elderly persons without chronic kidney disease: the health, aging, and body composition study Kidney Int 2007 71 3 239 44 10.1038/sj.ki.5002042 17183246

[B16] Singh D Whooley MA Ix JH Ali S Shlipak MG Association of cystatin C and estimated GFR with inflammatory biomarkers: the Heart and Soul Study Nephrol Dial Transplant 2007 22 4 1087 92 10.1093/ndt/gfl744 PMC2770338 17210589

[B17] Bild DE Bluemke DA Burke GL Detrano R Diez Roux AV Folsom AR Multi-ethnic study of atherosclerosis: objectives and design Am J Epidemiol 2002 156 9 871 81 10.1093/aje/kwf113 12397006

[B18] Stevens LA Coresh J Schmid CH Feldman HI Froissart M Kusek J Estimating GFR using serum cystatin C alone and in combination with serum creatinine: a pooled analysis of 3,418 individuals with CKD Am J Kidney Dis 2008 51 3 395 406 10.1053/j.ajkd.2007.11.018 PMC2390827 18295055

[B19] Murthy K Stevens LA Stark PC Levey AS Variation in the serum creatinine assay calibration: a practical application to glomerular filtration rate estimation Kidney Int 2005 68 4 1884 7 10.1111/j.1523-1755.2005.00608.x 16164667

[B20] Levey AS Stevens LA Schmid CH Zhang YL Castro AF Feldman HI A new equation to estimate glomerular filtration rate Ann Intern Med 2009 150 9 604 12 10.7326/0003-4819-150-9-200905050-00006 PMC2763564 19414839

[B21] Declerck PJ Alessi MC Verstreken M Kruithof EK Juhan-Vague I Collen D Measurement of plasminogen activator inhibitor 1 in biologic fluids with a murine monoclonal antibody-based enzyme-linked immunosorbent assay Blood 1988 71 1 220 5 3257145

[B22] Chobanian AV Bakris GL Black HR Cushman WC Green LA Izzo JL Jr The Seventh Report of the Joint National Committee on Prevention, Detection, Evaluation, and Treatment of High Blood Pressure: the JNC 7 report JAMA 2003 289 19 2560 72 10.1001/jama.289.19.2560 12748199

[B23] Nakano M Furutani M Shinno H Ikeda T Oida K Ishii H Elevation of soluble thrombomodulin antigen levels in the serum and urine of streptozotocin-induced diabetes model rats Thromb Res 2000 99 1 83 91 10.1016/s0049-3848(00)00216-4 10904105

[B24] Jacobson SH Egberg N Hylander B Lundahl J Correlation between soluble markers of endothelial dysfunction in patients with renal failure Am J Nephrol 2002 22 1 42 7 10.1159/000046673 11919402

[B25] Mezzano D Tagle R Pais E Panes O Perez M Downey P Endothelial cell markers in chronic uremia: relationship with hemostatic defects and severity of renal failure Thromb Res 1997 88 6 465 72 10.1016/s0049-3848(97)00280-6 9610957

[B26] Wu KK Soluble thrombomodulin and coronary heart disease Curr Opin Lipidol 2003 14 4 373 5 10.1097/00041433-200308000-00006 12865735

[B27] Breitenstein A Tanner FC Luscher TF Tissue factor and cardiovascular disease Circ J 2010 74 1 3 12 10.1253/circj.cj-09-0818 19996531

[B28] Toschi V Soluble tissue factor and tissue factor pathway inhibitor in cardiovascular disease J Thromb Haemost 2007 5 3 472 4 10.1111/j.1538-7836.2007.02402.x 17319902

[B29] Adams MJ Irish AB Watts GF Oostryck R Dogra GK Hypercoagulability in chronic kidney disease is associated with coagulation activation but not endothelial function Thromb Res 2008 123 2 374 80 10.1016/j.thromres.2008.03.024 18486198

[B30] Morange PE Blankenberg S Alessi MC Bickel C Rupprecht HJ Schnabel R Prognostic value of plasma tissue factor and tissue factor pathway inhibitor for cardiovascular death in patients with coronary artery disease: the AtheroGene study J Thromb Haemost 2007 5 3 475 82 10.1111/j.1538-7836.2007.02372.x 17204132

[B31] Folsom AR Aleksic N Park E Salomaa V Juneja H Wu KK Prospective study of fibrinolytic factors and incident coronary heart disease: the Atherosclerosis Risk in Communities (ARIC) Study Arterioscler Thromb Vasc Biol 2001 21 4 611 7 10.1161/01.atv.21.4.611 11304480

[B32] Wannamethee SG Shaper AG Lowe GD Lennon L Rumley A Whincup PH Renal function and cardiovascular mortality in elderly men: the role of inflammatory, procoagulant, and endothelial biomarkers Eur Heart J 2006 27 24 2975 81 10.1093/eurheartj/ehl402 17132648

[B33] Mannucci PM von Willebrand factor: a marker of endothelial damage? Arterioscler Thromb Vasc Biol 1998 18 9 1359 62 10.1161/01.atv.18.9.1359 9743222

[B34] Danesh J Wheeler JG Hirschfield GM Eda S Eiriksdottir G Rumley A C-reactive protein and other circulating markers of inflammation in the prediction of coronary heart disease N Engl J Med 2004 350 14 1387 97 10.1056/NEJMoa032804 15070788

[B35] Rumley A Lowe GD Sweetnam PM Yarnell JW Ford RP Factor VIII, von Willebrand factor and the risk of major ischaemic heart disease in the Caerphilly Heart Study Br J Haematol 1999 105 1 110 6 10233372

[B36] Bash LD Erlinger TP Coresh J Marsh-Manzi J Folsom AR Astor BC Inflammation, hemostasis, and the risk of kidney function decline in the Atherosclerosis Risk in Communities (ARIC) Study Am J Kidney Dis 2009 53 4 596 605 10.1053/j.ajkd.2008.10.044 PMC2778194 19110358

[B37] Folsom AR Delaney JA Lutsey PL Zakai NA Jenny NS Polak JF Associations of factor VIIIc, D-dimer, and plasmin-antiplasmin with incident cardiovascular disease and all-cause mortality Am J Hematol 2009 84 6 349 53 10.1002/ajh.21429 PMC2950108 19472201

[B38] Morange PE Bickel C Nicaud V Schnabel R Rupprecht HJ Peetz D Haemostatic factors and the risk of cardiovascular death in patients with coronary artery disease: the AtheroGene study Arterioscler Thromb Vasc Biol 2006 26 12 2793 9 10.1161/01.ATV.0000249406.92992.0d 17023678

[B39] Cushman M Lemaitre RN Kuller LH Psaty BM Macy EM Sharrett AR Fibrinolytic activation markers predict myocardial infarction in the elderly. The Cardiovascular Health Study Arterioscler Thromb Vasc Biol 1999 19 3 493 8 10.1161/01.atv.19.3.493 10073948

[B40] Shrivastava S Ridker PM Glynn RJ Goldhaber SZ Moll S Bounameaux H D-dimer, factor VIII coagulant activity, low-intensity warfarin and the risk of recurrent venous thromboembolism J Thromb Haemost 2006 4 6 1208 14 10.1111/j.1538-7836.2006.01935.x 16706961

[B41] Wattanakit K Cushman M Chronic kidney disease and venous thromboembolism: epidemiology and mechanisms Curr Opin Pulm Med 2009 15 5 408 12 10.1097/MCP.0b013e32832ee371 PMC2811494 19561505

[B42] Folsom AR Lutsey PL Astor BC Wattanakit K Heckbert SR Cushman M Chronic kidney disease and venous thromboembolism: a prospective study Nephrol Dial Transplant 2010 10.1093/ndt/gfq179 PMC2948836 20353958

[B43] Ishii H Nakano M Tsubouchi J Ishikawa T Uchiyama H Hiraishi S Establishment of enzyme immunoassay of human thrombomodulin in plasma and urine using monoclonal antibodies Thromb Haemost 1990 63 2 157 62 2163551

[B44] Lwaleed BA Chisholm M Francis JL Development and validation of an assay for urinary tissue factor activity J Clin Pathol 1999 52 3 219 24 10.1136/jcp.52.3.219 PMC501083 10450183

